# Deletion of *Cd151* reduces mammary tumorigenesis in the MMTV/PyMT mouse model

**DOI:** 10.1186/1471-2407-14-509

**Published:** 2014-07-11

**Authors:** Séverine Roselli, Richard GS Kahl, Ben T Copeland, Matthew J Naylor, Judith Weidenhofer, William J Muller, Leonie K Ashman

**Affiliations:** 1 School of Biomedical Sciences and Pharmacy, Faculty of Health and Medicine, Priority Research Centre in Cancer, University of Newcastle, Newcastle, NSW, Australia; 2 Hunter Medical Research Institute, Cancer Research Program, Newcastle, NSW, Australia; 3 School of Medical Sciences, University of Sydney, Sydney, NSW, Australia; 4 Goodman Cancer Centre, McGill University, Montreal, Canada

**Keywords:** Tetraspanin, CD151, Breast, Cancer, Metastasis

## Abstract

**Background:**

Tetraspanins are transmembrane proteins that serve as scaffolds for multiprotein complexes containing, for example, integrins, growth factor receptors and matrix metalloproteases, and modify their functions in cell adhesion, migration and transmembrane signaling. CD151 is part of the tetraspanin family and it forms tight complexes with β1 and β4 integrins, both of which have been shown to be required for tumorigenesis and/or metastasis in transgenic mouse models of breast cancer. High levels of the tetraspanin CD151 have been linked to poor patient outcome in several human cancers including breast cancer. In addition, CD151 has been implicated as a promoter of tumor angiogenesis and metastasis in various model systems.

**Methods:**

Here we investigated the effect of *Cd151* deletion on mammary tumorigenesis by crossing *Cd151*-deficient mice with a spontaneously metastasising transgenic model of breast cancer induced by the polyoma middle T antigen (PyMT) driven by the murine mammary tumor virus promoter (MMTV).

**Results:**

*Cd151* deletion did not affect the normal development and differentiation of the mammary gland. While there was a trend towards delayed tumor onset in *Cd151*^−/−^ PyMT mice compared to *Cd151*^+/+^ PyMT littermate controls, this result was only approaching significance (Log-rank test *P*-value =0.0536). Interestingly, *Cd151* deletion resulted in significantly reduced numbers and size of primary tumors but did not appear to affect the number or size of metastases in the MMTV/PyMT mice. Intriguingly, no differences in the expression of markers of cell proliferation, apoptosis and blood vessel density was observed in the primary tumors.

**Conclusion:**

The findings from this study provide additional evidence that CD151 acts to enhance tumor formation initiated by a range of oncogenes and strongly support its relevance as a potential therapeutic target to delay breast cancer progression.

## Background

Breast cancer is the most commonly diagnosed cancer among women and despite some major advances in diagnosis and treatment, it remains the second leading cause of cancer death in women worldwide. Similarly to other cancers, some of the major challenges in the treatment of breast cancer reside in the lack of response or development of resistance to existing therapies and the devastating consequences of metastasis. Better prognostic markers as well as new targeted treatments that could be used alone, or most likely in combination with existing therapies are needed to improve patient outcomes.

The tetraspanin CD151 is part of the tetraspanin family of transmembrane proteins, which consists of 33 members in humans. These proteins serve as scaffolds for multiprotein complexes (called TEMs or Tetraspanin-Enriched Microdomains) where they associate with molecules such as integrins, growth factor receptors and matrix metalloproteases, modifying their functions in various cellular processes. CD151 forms tight complexes with the laminin binding integrins (α3β1, α6β1 and α6β4) [1], modulates their signaling and contributes to integrin mediated cell adhesion and motility.

Interestingly, β1-integrin and β4-integrin- heterodimers (reviewed in [2]) are both expressed by breast epithelial cells (mostly as α3β1 and α6β4) and have been shown to be required for tumorigenesis and metastasis in the MMTV/PyMT and MMTV/Neu (rat homolog of ErbB2) mouse models of breast cancer [3,4], respectively. CD151 expression itself has been associated with poor patient outcome in several malignancies, including cancers of the breast [5,6], prostate [7], lung [8], and kidney [9], whereas it has also been found to correlate with improved survival in endometrial cancer [10]. Notably in breast cancer, elevated expression of CD151 correlates with lymph node invasion and poor overall survival of patients with invasive ductal carcinoma [5,6]. In accordance with its association with poor prognostic in patients, CD151 has been implicated as a promoter of tumor angiogenesis and/or metastasis *in vitro* in human breast cancer cell lines and in several *in vivo* model systems including xenografts [5,11], matrigel plug and tumor implantation experiments [12], as well as experimental metastasis models [13,14]. In addition, a recent study showed that CD151 plays a role in mammary cell proliferation, suggesting the involvement of CD151 in tumor cell growth [15].

Altogether these data strongly indicate a role for CD151 in tumor growth and metastasis, suggesting that it could be used as a target molecule for the design of new breast cancer therapies. However, when we started this work, the possible direct cause-effect relationship between CD151 expression and breast tumor onset/progression and metastasis had never been tested. In order to address this question, we studied the effect of *Cd151* deletion on *de novo* breast tumorigenesis and spontaneous metastasis in the very well characterized MMTV/PyMT transgenic breast cancer mouse model [16]. In this model, the polyoma middle T oncogene is expressed under the transcriptional control of the mouse mammary tumor virus promoter. The mammary tumors that develop in MMTV/PyMT female mice recapitulate the histological stages of human breast cancer from premalignant lesions to invasive carcinoma [17] and they also display activation of the same signaling pathways that act downstream of the ErbB2 oncogene and are often activated in breast cancer, such as c-Src, PI3K and Ras [18].

It is interesting to note that a study addressing the impact of *Cd151* deletion in another mouse model of breast cancer (the ErbB2 model) has recently been published by Deng and colleagues [19]. The results from both studies will be compared in the discussion.

Here we show that *Cd151*–null mice develop smaller and fewer PyMT mammary tumors than their age matched controls. Our results suggest that *Cd151* deletion impairs tumor initiation and/or tumor growth in the MMTV/PyMT model, while an apparent effect on tumor metastasis could be attributable to larger tumor burden in *Cd151*^*+/+*^ mice.

## Methods

### Experimental animals

Animal maintenance was in accordance with the Animal Care and Ethics Committee at the Australian BioResources specific pathogen free (SPF) animal breeding facility (Moss Vale, New South Wales). All animal monitoring and experiments were approved by the Animal Care and Ethics Committee at the University of Newcastle. In the tumorigenesis experiments, we used the well-characterized FVB/N (FVB) MMTV/PyMT mouse line (MT#634) carrying a mouse mammary tumor virus promoter-driven polyoma middle T transgene [16]. A pure FVB genetic background is most commonly used for mouse tumorigenesis experiments because of its permissiveness to spontaneous tumor development. *Cd151*^−/−^ mice are grossly healthy on the C57Bl/6 (B6) background [20] but they develop a severe kidney disease on a pure FVB background [21]. Hence FVB *Cd151*^−/−^ mice could not be used for the tumorigenesis experiments. Since F1 hybrid FVB x B6 *Cd151*^−/−^ mice were healthy and did not present any sign of kidney disease onset (monitored for the appearance of proteinuria over a 12 months period, our unpublished data), we conducted the experiments on this hybrid background. This allowed us to keep all experimental animals on a mixed but yet homogenous (50% B6 and 50% FVB) genetic background. FVB *Cd151*^+/−^ mice were produced by backcross for 10 generations from the original B6 *Cd151*^−/−^[20] and maintained as heterozygotes. Heterozygous B6 *Cd151*^+/−^ females were crossed with FVB *Cd151*^+/−^ males carrying the MMTV/PyMT transgene (PyMT *Cd151*^+/−^ males, see Additional file 1: Figure S1 for breeding details) in order to generate the experimental F1 animals. Genotyping was performed as previously described [20]. Experimental and control littermates were co-housed throughout the experiments, in a temperature controlled facility with a 12-h light: dark cycle.

### Animal monitoring and tissue collection

Beginning at weaning (3 weeks of age), female mice were palpated twice weekly for the onset of mammary tumors. For each mouse, tumor palpation was performed in each of the ten mammary glands, in a genotype-blinded fashion. At 15–16 weeks of age, female mice were euthanased by CO_2_ inhalation, and all the tumors were dissected and weighed. For each experimental mouse, half of the biggest tumor was fixed in 10% neutral buffered formalin (NBF) for paraffin embedding, one quarter was snap frozen in liquid nitrogen, and the last quarter was snap frozen in OCT compound. At the time of dissection and after excision of the tumors, the lungs were exposed and inflated via tracheal injection of 1 ml of 10% NBF in order to inflate and fix the lung lobes. Lungs were then excised and further fixed in 10% NBF for at least 24 hours before paraffin embedding.

### Whole mount analysis

Whole mount analysis was performed by spreading inguinal #4 mammary glands onto poly-lysine slides followed by overnight fixation in 10% NBF, defatting in acetone and overnight staining in carmine alum (0.2% carmine and 0.5% aluminium sulphate) as previously described [22]. The stained glands were then dehydrated in a graded ethanol series, incubated in xylene for 1 hour and stored in methyl salicylate.

### Tumor histology and immunohistochemistry

Tumor histology/stage was assessed on the largest tumor for each mouse using 5 μm paraffin sections stained with hematoxylin and eosin. Immunohistochemistry (IHC) was performed on 5 μm paraffin sections using a peroxidase VECTASTAIN ABC elite kit and DAB peroxidase substrate kit as per the manufacturer’s recommendations (Vector Laboratories, Burlingame, CA). The antibodies and dilutions used for IHC were rabbit monoclonal anti-Ki67 at 1:200 (Neomarkers, Kalamazoo, MI, USA) and polyclonal rabbit anti-cleaved caspase 3 at 1:800 (Cell Signaling Technology, Danvers, MA, USA).

### Immunofluorescence labeling

Immunofluorescence labelings were performed on 5 μm frozen sections as previously described [21]. Primary antibodies and dilutions used for immunofluorescence labeling were rabbit anti-CD151 (LAI-2) at 1:500 [21]; rat anti-CD31 (BD Biosciences, Franklin Lakes, NJ, USA) at 1:200; rabbit anti-α3 integrin (a kind gift from Dr. Fiona Watt, Wellcome Trust Centre for Stem Cell Research, Cambridge, UK) at 1:1000; rat anti-β1 integrin (BD Biosciences) at 1:200; rat anti-β4 integrin (BD Biosciences) at 1:200; rat anti- α6 integrin (Chemicon, Temecula, CA, USA) at 1:200. The double labeling where two rabbit primary antibodies were used (CD151 and α3 integrin) was performed sequentially following established methods as described previously [21].

### Quantitation of tumor cell proliferation, apoptosis and vascularization

The largest tumor for each mouse was used to quantitate proliferation, apoptosis and vascularization in a genotype-blinded manner. Tumor cell proliferation was assessed by immunohistochemistry (as described above) for the commonly used Ki67 nuclear marker. Ki67 stained slides were scanned in a digital format using the Aperio™ digital pathology system (Aperio) and 200× magnification snapshots of digital images were generated using Imagescope. Quantitation of proliferation (expressed as percent Ki67 positive nuclear area per total nuclear area) was then performed on the 200× digital images using ImmunoRatio, a publicly available web-based application [23]. The extent of apoptosis in the tumors was quantitated on Aperio images of cleaved-caspase 3 IHC stained slides, using the positive pixel count algorithm at 200× magnification (5 fixed size (300 μm × 300 μm) images were used for each tumor section).

To estimate blood vessel density, CD31 immunofluorescence labeling was performed and Image J was used to quantitate the proportion of CD31 positive area inside the tumors. At least five fluorescent microscope pictures (100× magnification) per tumor were used in the CD31 analysis.

### Analysis of lung metastasis

All the lung lobes were dissected and processed for paraffin embedding. Five microns paraffin sections were stained with hematoxylin and eosin and slides were scanned in a digital format using the Aperio™ digital pathology system (Aperio). The lung area per section was measured using Scanscope and the metastatic burden (mm^2^ of metatastases/cm^2^ lung) was calculated for each animal, using the data from 3 sections at least 100 μm apart, as previously described [24].

### Statistical analysis

Statistical analysis was conducted using Prism 6 (Graphpad software). Kaplan-Meier survival curves were analysed with the log-rank test. Metastasis distribution was assessed with a contingency table and Chi-square test. All the other data sets were submitted to the Shapiro-Wilk normality test and depending on the result of this test, parametric or non-parametric comparison tests were performed. Specific tests used for each data set are mentioned in the figure legends. In all tests, *P*-values <0.05 were considered significant.

## Results

### Deletion of *Cd151* does not interfere with mammary gland development and differentiation

**Figure 1:**
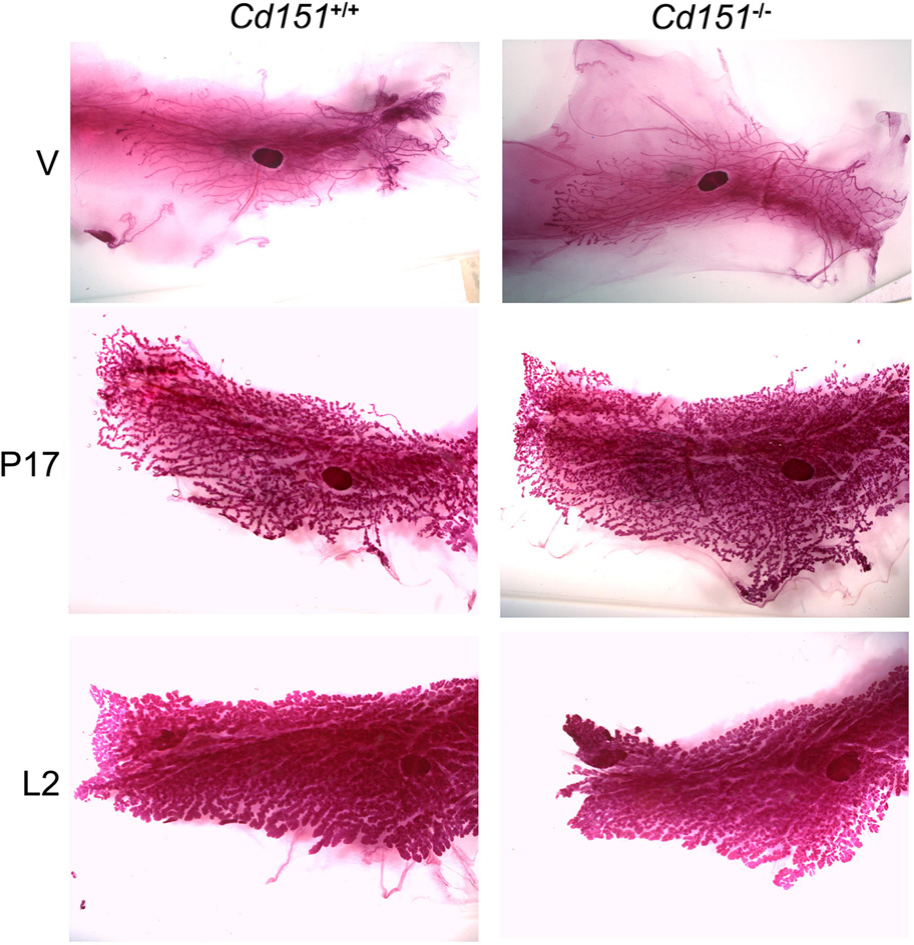
**Whole mount analysis of mammary gland development in *Cd151***^**+/+ **^**and *Cd151 ***^**−/− **^**mice.** The morphology of whole mount #4 mammary glands was analysed at different stages in FVB *Cd151*^+/+^ and *Cd151*^−/−^ mice. Mammary gland development was assessed in virgin glands (V, top panel) and appeared to occur normally in *Cd151*^−/−^ animals as compared to *Cd151*^+/+^ mice. Mammary gland differentiation during pregnancy (day 17 of pregnancy, middle panel) and lactation (lactation day 2, bottom panel) was also unchanged in *Cd151*^−/−^ mice.

Before investigating the effect of *Cd151* deletion on mammary tumorigenesis it was necessary to determine whether mammary gland development and differentiation was normal in *Cd151* knock-out mice. For this purpose, we performed whole mount analysis of #4 inguinal mammary glands in virgin, pregnant and lactating FVB *Cd151*^+/+^ and *Cd151*^−/−^ mice (Figure 1). Ductal outgrowth and mammary gland differentiation appeared grossly normal in the *Cd151*^−/−^ females at the different stages of development investigated.

### Expression pattern of CD151 in the normal mammary gland and PyMT mammary tumors

**Figure 2:**
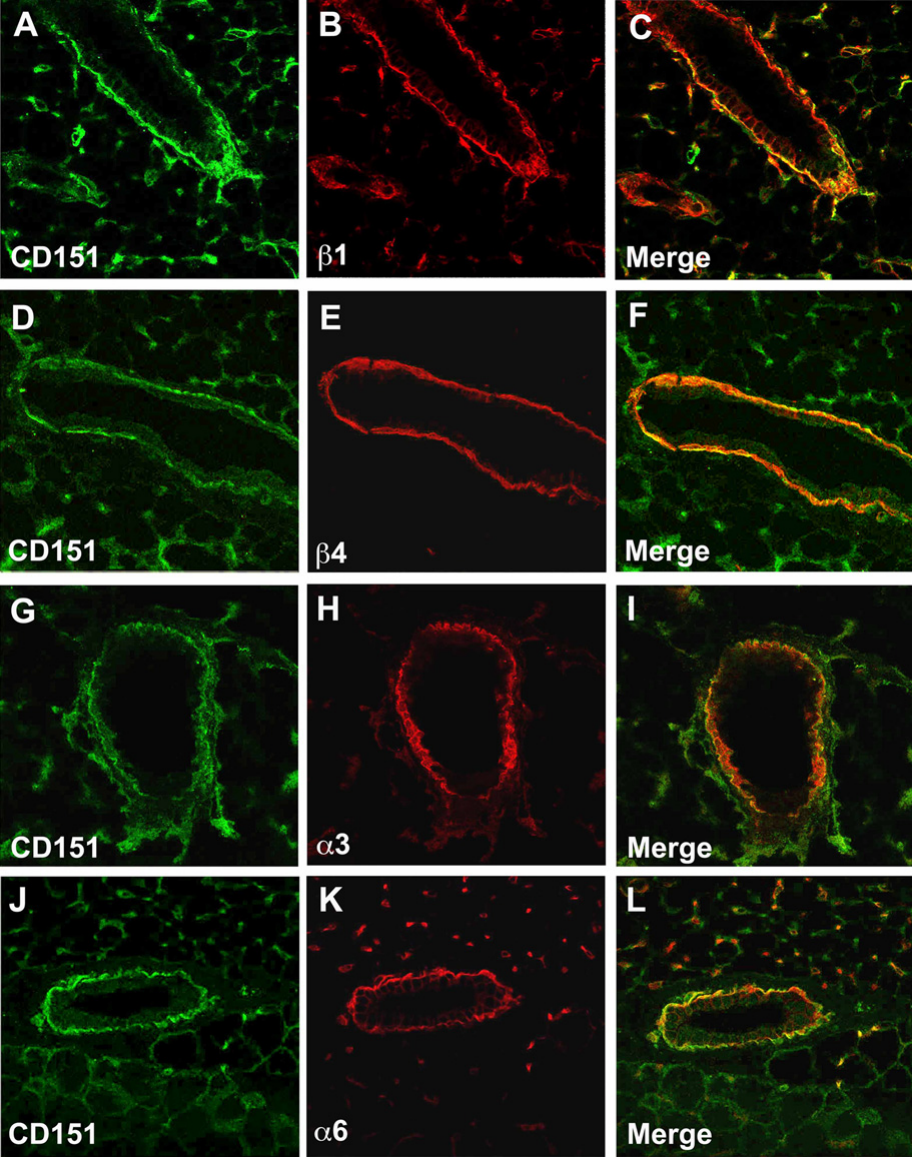
**Localization of CD151 in the wild-type mouse mammary gland.** Dual immunofluorescent labeling and confocal analysis using CD151 antibodies (green) and antibodies towards the integrin chains β1 **(A**-**C)**, β4 **(D**-**F)**, α3 **(G**-**I)** and α6 **(J**-**L)** in red. CD151 co-localizes with these 4 chains of integrins in the basal cell layer of the mammary epithelium and the basolateral membrane of luminal epithelial cells. Original magnification ×400.

**Figure 3:**
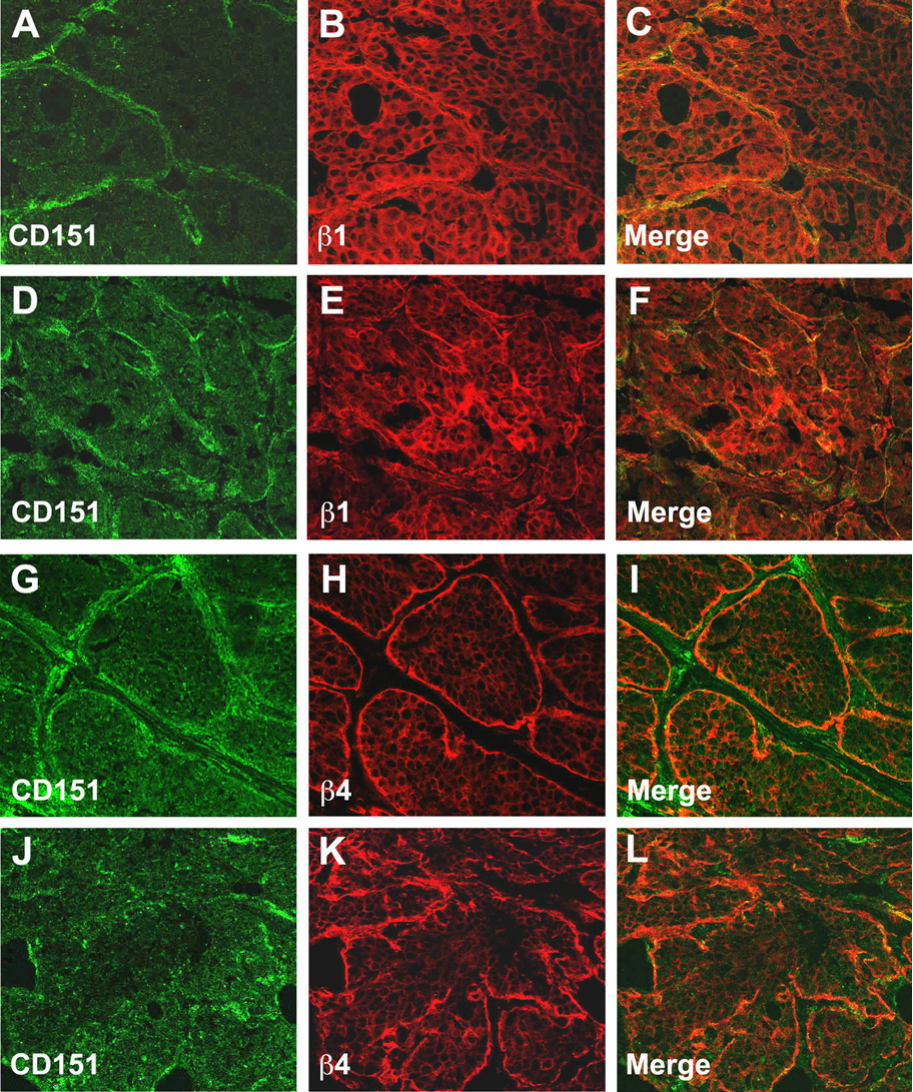
**Expression of CD151 in mammary tumors.** Dual immunofluorescent labeling and confocal analysis using CD151 antibodies (green) and antibodies towards β1-**(A**-**F)**, or β4-integrin **(G**-**L)** were performed on *Cd151*^+/+^ MMTV/PyMT mammary tumors at the adenoma stage **(A**-**C and G**-**I)** and the carcinoma stage **(D**-**F and J**-**L)** (stages as defined by Lin et al. [17]). At the early adenoma stage **(A**-**C and G**-**I)**, the tumor cells showed faint CD151 expression overall that was more intense on the edge of the tumor nodules, colocalizing with β1 and β4 integrins along the basement membrane. At a more advanced tumor stage, the labeling was more patchy with still some degree of colocalization with integrins **(D**-**F and J**-**L)**. Original magnification ×400.

Immunofluorescence labeling of 6-week old virgin mammary glands and confocal microscopy revealed strong expression of CD151 in the mammary ducts in a basolateral pattern (Figure 2), reminiscent of the surrounding basal layer of myoepithelial cells and in accordance with what has been previously reported in humans [11]. Fainter CD151 labeling was also noticeable in the basolateral membrane of ductal luminal cells (Figure 2A). Moreover, CD151 labeling in the mammary ducts colocalized to some extent with the α3-, α6-, β1- and β4- chains of integrins, suggesting association of CD151 with the α3β1, α6β1 and α6β4 integrin heterodimers in the mammary gland. Similarly to the situation in the normal glands, CD151 appeared to be expressed strongly by the myoepithelial cell layer surrounding the tumor nodules of the early stage PyMT tumors, while the cancer cells in the nodules were only faintly labeled (Figure 3). The CD151 labeling overlapped to some extent with the β1- and β4- integrin immunolabelings in the tumors suggesting as expected some degree of colocalization.

### Loss of *Cd151* significantly decreases mammary tumor multiplicity and growth

To avoid the kidney phenotype that has been described in FVB *Cd151*^−/−^ mice [21], we chose to investigate the effects of *Cd151* deletion on mammary tumorigenesis on a F1 (FVB×B6) background (see explanations in Methods). Consistent with previous reports showing increased PyMT tumor latency on the B6 background [24,25], the onset of mammary tumors was significantly delayed by about 20 days on the (FVBxB6) F1 background in comparison to the pure FVB background (Additional file 2: Figure S2).

**Figure 4:**
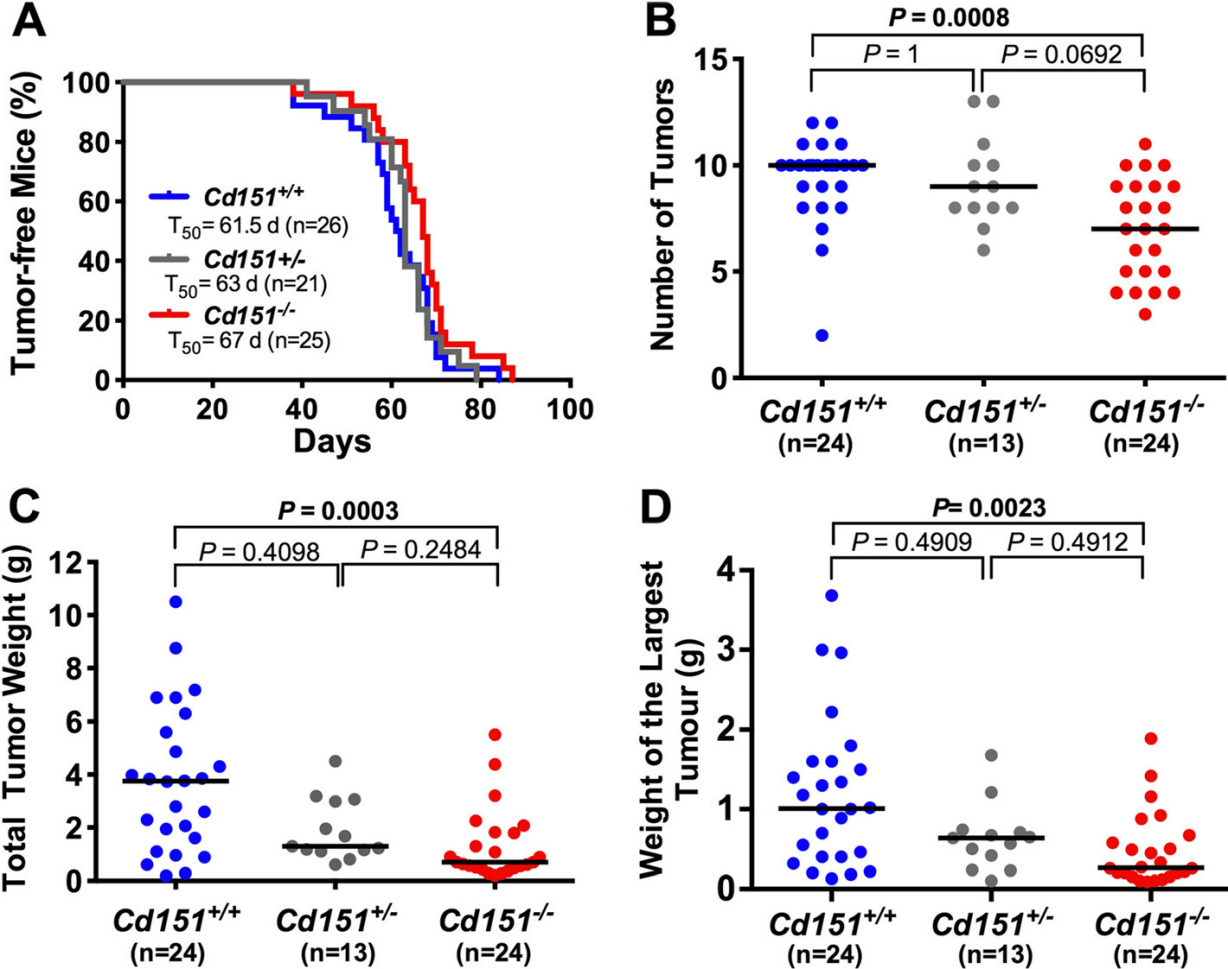
**Effect of *Cd151 *deletion on MMTV/PyMT tumor onset and growth. (A)** The appearance of mammary tumors was monitored in MMTV/PyMT *Cd151*^+/+^, *Cd151*^+/−^ and *Cd151*^−/−^ mice by bi-weekly palpation and is represented on a Kaplan-Meier survival curve. T_50_ indicates the median time to development of a palpable mammary tumor for each group of mice. The differences in T_50_ between *Cd151*^+/+^ and *Cd151*^−/−^ groups or between the *Cd151*^+/+^ and the *Cd151*^+/−^ groups were not statistically significant (log rank test *P =* 0.0536 and *P* = 0.7533, respectively). Tumor multiplicity **(B)**, total tumor weight **(C)** and the weight of the biggest tumor per mouse **(D)** were measured at the time of euthanasia. The horizontal bars on the graphs represent the median for each parameter. The results were compared using the non-parametric Krustal-Wallis test. The number of tumors (**B**, *P* = 0.0003), the total tumor weight per mouse (**C**, *P* = 0.0002) and the weight of the biggest tumor per mouse (**D**, P = 0.0014) were significantly decreased in the *Cd151*^−/−^ group compared to the *Cd151*^+/+^. mice. There were no significant differences when comparing *Cd151*^+/+^ with *Cd151*^+/−^ groups or *Cd151*^+/−^ with *Cd151*^−/−^ respectively.

Investigation of mammary tumor onset by palpation showed no statistically significant difference in tumor latency between *Cd151*^+/+^ and *Cd151*^−/−^ PyMT mice (Figure 4A). There was however a trend towards increased tumor latency in *Cd151*^−/−^ (T_50_ = 67 days; n = 25) as compared to *Cd151*^+/+^ (T_50_ = 61.5 days; n = 26) that was approaching significance (Log-rank test *P*-value =0.0536). Interestingly, the median age to tumor onset in the heterozygous *Cd151*^*+/−*^ mice was in between the values reported for *Cd151*^+/+^ and *Cd151*^−/−^ PyMT mice (*Cd151*^+/−^ T_50_ = 63, n = 21). However, the difference between the median tumor latency of *Cd151*^+/−^ versus *Cd151*^+/+^ mice was not significant (Log-rank test *P* = 0.7533).

**Figure 5:**
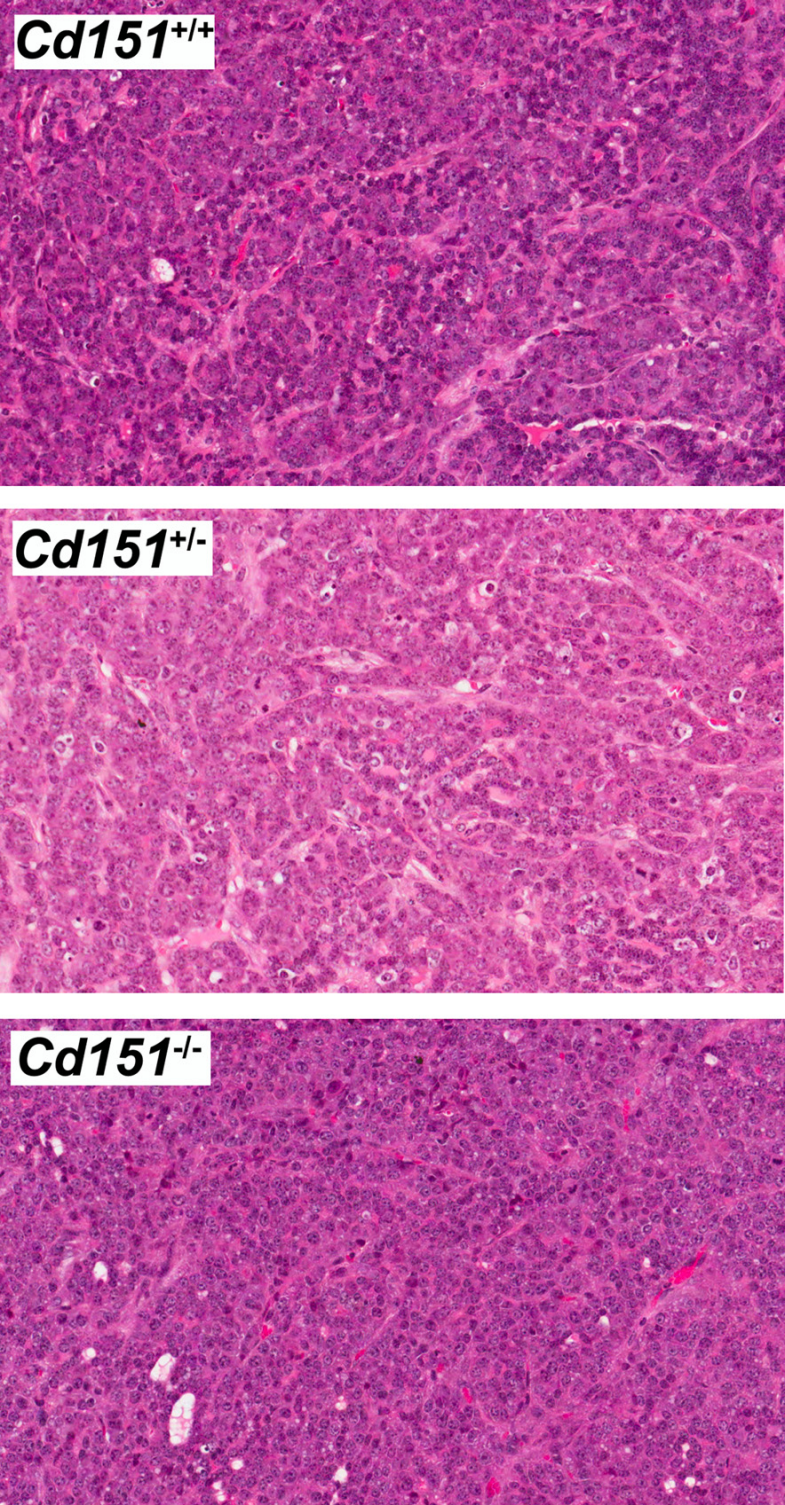
**Tumor histology of the MMTV/PyMT *Cd151***^**+/+**^**, *Cd151***^**+/− **^**and *Cd151***^**−/− **^**mice.** At the end point, tumor histology (assessed by H&E staining) was undistinguishable between the three groups of mice. All mammary tumors presented features of the carcinoma stage, independently of their *Cd151* genotype.

**Figure 6:**
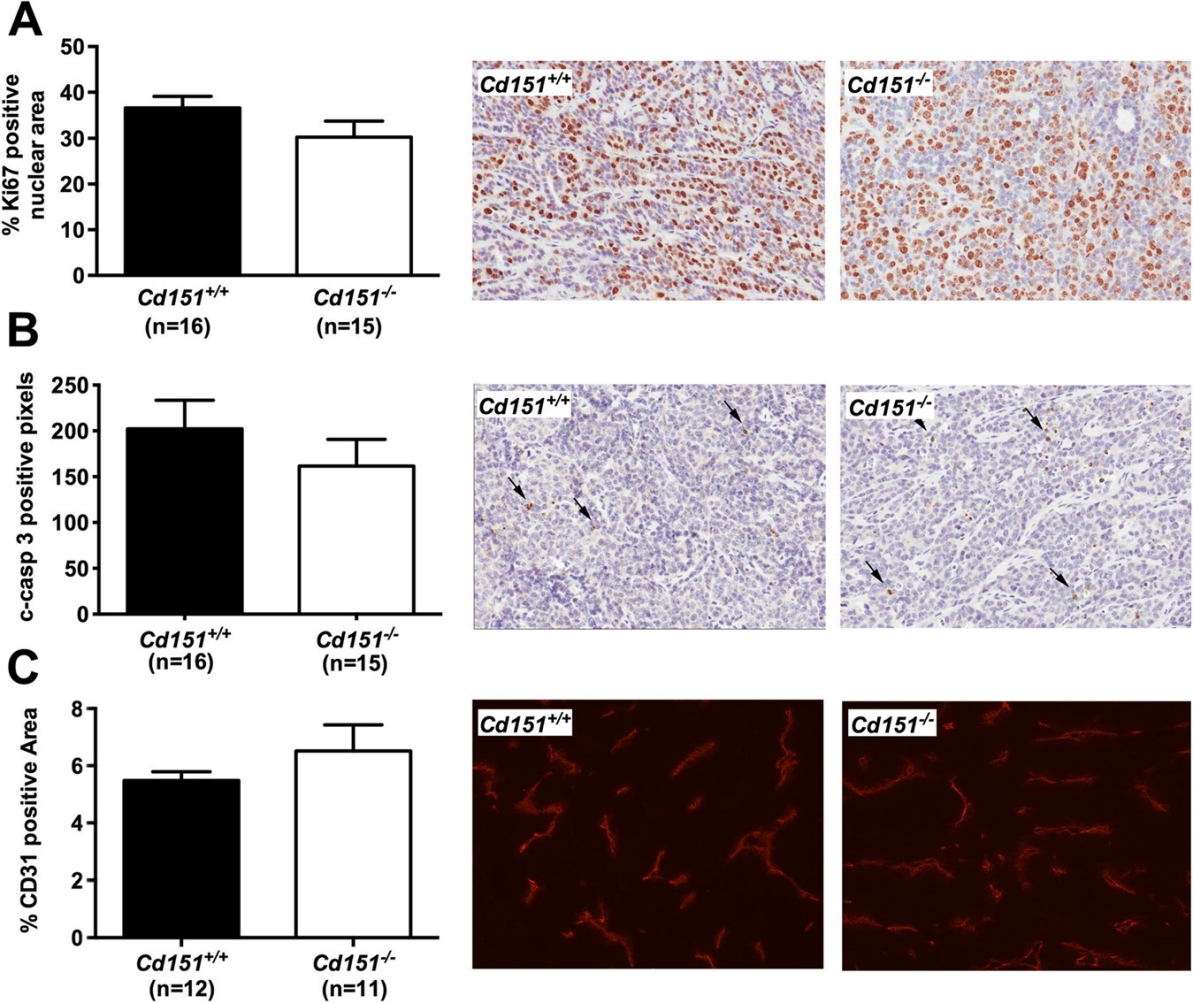
**Tumor cell proliferation, apoptosis and blood vessel density in *Cd151***^**+/+ **^**and *Cd151***^**−/− **^**mammary tumors.** These parameters were assessed by IHC for the cell proliferation marker Ki67 (**A**, *P* =0.1533), the apoptosis marker cleaved-caspase 3 (**B**, *P* =0.3476), and by immunofluorescence for the endothelial cell specific antigen CD31 (**C**, *P* = 0.3035), respectively. Means ± SEM are represented and parametric t-test was used to compare the data. Representative photos of the immunolabelings are also shown. Original magnification: ×200 for Ki67 and Cleaved-caspase 3 and ×100 for CD31.

At 15 to 16 weeks of age (i.e. 6 to 7 weeks after initial tumor palpation), the mice were euthanased and all the tumors were collected and weighed for each mouse. Interestingly both the total tumor weight (*P* = 0.0003) and tumor multiplicity (*P* = 0.0008) per mouse were significantly decreased in the *Cd151*^−/−^ group as compared to *Cd151*^+/+^ group (Figure 4B and 4C). Individual tumor weight (as shown with weight of the largest tumor per mouse Figure 4D) was also clearly decreased in *Cd151*^−/−^ mice (*P* = 0.0023), suggesting an effect of *Cd151* gene deletion on both mammary tumor induction and growth/proliferation *in vivo*. Regardless of the *Cd151* genotype, at least part of the largest tumor of each mouse had progressed to the carcinoma stage as determined by H&E staining (illustrated in Figure 5). Immunolabelings for Ki67, cleaved caspase-3 and CD31 were performed on representative primary tumor samples in order to compare the rates of cell proliferation, apoptosis and tumor angiogenesis between both groups of mice, respectively (Figure 6). These experiments did not reveal any significant differences between *Cd151*^+/+^ and *Cd151*^−/−^ tumors.

### Effect of *Cd151* deletion on development of lung metastases

**Figure 7:**
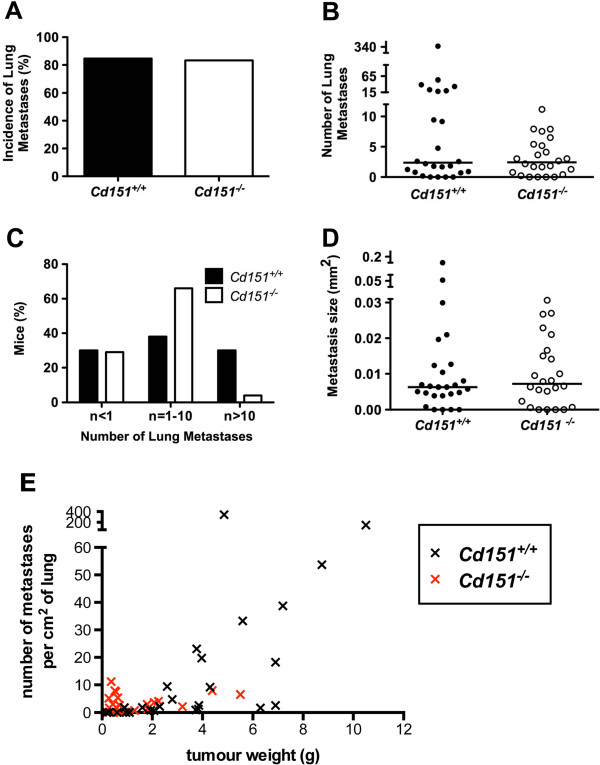
**Impact of *Cd151 *deletion on lung metastases.** The incidence of lung metastasis **(A)**, the median number of lung metastases (per cm^2^) per mouse (**B**, *P* = 0.3554), and the average metastasis size per mouse (mm^2^) (**D**, *P* = 0.6757) were not different between *Cd151*^+/+^ (n = 26) and *Cd151*^−/−^ (n = 24) mice. Non-parametric Mann–Whitney test was used to compare the data in B and D and the horizontal bars indicate the median values. The distribution of the number of metastases per mouse was much more heterogenous in the *Cd151*^+/+^ group **(C)**: A significantly larger proportion of *Cd151*^+/+^ (8/26) than *Cd151*^−/−^ (1/24) mice had 10 or more metastatic foci per cm^2^ of lung (Chi square *P* value = 0.0329). **(E)** Correlation between the number of metastases and the total tumor weight per mouse was assessed using the non parametric Spearman correlation test. The number of metastases did not correlate with tumor burden in the *Cd151*^*−/−*^ group (r = 0.117, *P =* 0.5861). There was however a strong correlation between number of metastases and tumor weight in the *Cd151*^*+/+*^ group (r = 0.8046, *P* < 0.0001), which contains mice with larger tumor burden than the *Cd151*^*−/−*^ group.

Since the mammary tumors of MMTV/PyMT mice primarily metastasize to the lungs, we examined the lungs of *Cd151*^+/+^ and *Cd151*^−/−^ PyMT mice at the time of dissection, which corresponds to 6 to 7 weeks after tumor onset. The incidence of lung metastasis was similar in both groups with 22 out of 26 *Cd151*^+/+^ (85%) and 20 out of 24 *Cd151*^−/−^ (83%) mice developing lung metastases (Figure 7A). In addition, the median number of lung metastases (Mann Whitney test *P*-value = 0.3554) and the average size of individual metastasis per mouse (Mann Whitney test *P*-value = 0.6757) were similar between the two groups (Figure 7B and 7D, respectively). The distribution of the number of metastases in the *Cd151*^+/+^ group, was much more heterogeneous (Figure 7B), with a large subset of the *Cd151*^+/+^ but not *Cd151*^−/−^ group of mice containing high numbers of metastases (Chi-square *P*-value = 0.0329, Figure 7C). On closer observation, it could be noted that the mice with high number of metastases in the *Cd151*^+/+^ group were also the mice with higher tumor burden (Figure 7E) suggesting that tumour burden rather than *Cd151* status is the likely explanation. In conclusion, ablation of *Cd151* per se did not appear to affect the number or size of metastases in the MMTV/PyMT mice.

## Discussion

In this study, we found that deletion of *Cd151* significantly impaired tumor development and progression in the murine MMTV/PyMT breast tumorigenesis model. These results are consistent with previous work investigating CD151’s function in other breast tumor models, suggesting that regardless of the tumor initiating oncogene, CD151 enhances tumor initiation and subsequent progression. Whilst the increase in tumor latency in *Cd151*^−/−^ mice was only approaching significance, there was significant decrease in the number of tumors per mouse, suggesting that *Cd151* deletion might impair PyMT tumor initiation. This could be determined in future experiments by assessing the early stages of PyMT tumorigenesis (from the age of weaning) and whether the extent of hyperplasia is different between genotypes before a palpable tumor can be detected. The decreased number of detectable tumors could also reflect a pronounced defect in tumor growth. Indeed, the size of the mammary tumors was also reduced in *Cd151*^−/−^ mice, as shown by the total tumor burden per mouse (expressed as total tumor weight per mouse), as well as the individual tumor weight (represented as the weight of the largest tumor for each mouse). Intriguingly however we did not find a difference in tumor vascularisation or tumor cell proliferation and apoptosis that could explain the decreased tumor size in *Cd151*^*−/−*^ mice. In conclusion, the absence of effect on tumor proliferation and apoptosis reinforces the hypothesis stated above that *Cd151* deletion may act predominantly by delaying PyMT tumor initiation, as suggested by the trend towards delayed tumor onset and the decreased number of tumors per mouse.

In recent years, Sadej and colleagues have reported a decrease in tumor growth associated with CD151 knock-down in a subcutaneous xenograft model [5]. Similar to our result, CD151 knock-down did not have an effect on overall vessel density in the xenografts. Interestingly however the authors of that study described a pronounced decrease in the dense angiogenic network typically observed at the subcutaneous border of this type of tumors. We were not able to similarly assess the tumor border in our model system due to the nature of the *de novo* MMTV/PyMT tumors and because rich vascular networks do not develop at the periphery of tumors in this model.

Tumor progression in the MMTV/PyMT model has been very well characterized; it follows several well-defined stages from hyperplasia to non-invasive adenoma and finally invasive carcinoma [17]. Loss of the myoepithelial cell layer surrounding the hyperplastic luminal epithelial structures is an important step in the progression to invasive carcinoma in these tumors [26,17]. Moreover, mammary myoepithelial cells have been referred to as ‘natural tumor suppressors’ because of their capacity to block tumor cell growth and invasion [27,28]. Because CD151 is primarily expressed in the mammary myoepithelial cells and the PyMT tumors arise from luminal epithelial cells in the mammary gland where CD151 has reduced expression, it is tempting to speculate that *Cd151* deletion could affect tumor development indirectly through the microenvironment, by modifying the phenotype of the myoepithelial cells. However CD151 is also expressed, to a lesser extent, at the basolateral membrane of the luminal epithelium. It would be interesting to evaluate separately the specific effect of *Cd151* deletion in the stroma (tumor vasculature, immune cells, myoepithelial cells) and the luminal epithelium/tumor cells respectively, using a conditional knock-out model. In addition, one could study the tumor cell autonomous versus microenvironment effect by performing orthotopic tumor grafts.

A recent study [19] investigated the impact of *Cd151* deletion on tumor onset, growth and metastasis of another well characterized mouse mammary tumor model, the MMTV/Neu model (overexpressing multiple copies of wild-type Neu, the rat homolog of ErbB2). In their study, Deng and colleagues observed a significant delay in tumor onset as well as a decreased number of metastases per animal in the *Cd151*^−/−^ and *Cd151*^+/−^ groups compared with *Cd151*^+/+^ and they also suggest that the effects of CD151 in the MMTV/Neu model are largely mediated through α6β4 integrin. Together our data shows that *Cd151* deletion significantly impairs tumor occurrence and growth. On assessment of lung metastasis, we obtained different results from Deng and colleagues. The median incidence, number and size of lung metastases did not vary with the *Cd151* genotype in our study, and the higher metastatic burden in a proportion of wild-type mice was associated with increased primary tumors. These results suggest that ablation of *Cd151* does not directly affect metastasis in the PyMT model. Future studies using experimental metastasis models will be required to elucidate the contradictory effects on metastasis in the MMTV/Neu and MMTV/PyMT models.

In addition, two other studies have assessed the role of CD151 on *de novo* tumorigenesis in mouse cancer models other than breast. Firstly, similarly to our results in the PyMT breast cancer model, Takeda and colleagues have demonstrated that CD151 promotes tumor incidence and multiplicity in a skin carcinogenesis model [14]. Secondly, recent work in our laboratory has shown that deletion of *Cd151* reduces spontaneous metastasis of prostate tumors in the TRAMP model [29]. Altogether, the literature and the data we report here identify CD151 as an enhancer of *de novo* tumorigenesis and/or spontaneous metastasis across a broad range of cancer types.

Interestingly, the functional role of CD151 in tumorigenesis and metastasis has been mainly linked to its association with the laminin receptors in the literature. These adhesion receptors include mostly α3β1, α6β1 and α6β4 integrins in the mammary epithelium. Laminin binding integrins have a well-documented role in malignant cell processes regulating crucial functions such as cell proliferation and invasion. β1-integrins represent the most predominantly expressed integrins in the mammary epithelium and their direct involvement in mammary tumorigenesis has been extensively demonstrated using mouse models of breast cancer. For example, it has been shown that β1-integrin is absolutely required for PyMT mammary tumor initiation and progression [4,30]. Similarly, several studies have demonstrated that ablation of crucial signaling molecules downstream of β1-integrin such as focal adhesion kinase (FAK) also dramatically decreased cancer cell proliferation [31-33]. In contrast to the total block in PyMT induced tumorigenesis, mammary epithelial deletion of β1 integrin in an MMTV/Neu (activated ErbB2) mouse model did not totally prevent tumor development. Although it significantly delayed tumor onset, all of the mice developed tumors that were histologically identical to control mice [34]. The major effect of β1 deletion in the context of activated ErbB2 mammary tumorigenesis was the decrease in incidence of lung metastasis as well as decreased metastatic burden. There is to our knowledge no published report on the role of β4-integrin in the MMTV/PyMT model but it has been shown however that β4-integrin collaborates with ErbB2 to promote mammary tumorigenesis [3,16] in the MMTV/Neu mouse model.

## Conclusions

In summary, the results of this study show that CD151 enhances mammary tumor initiation and progression in the MMTV/PyMT mouse model but no direct effect on metastasis was demonstrated. The effects of *Cd151* deletion in the MMTV/PyMT model are likely to be mediated by a combination of both β1- and β4-integrins but this remains to be tested. In conclusion, our findings strongly support the relevance of CD151 as a potential therapeutic target to delay breast cancer progression.

## Abbreviations

IHC: Immunohistochemistry; NBF: Neutral buffered formalin; PyMT: Polyoma middle T antigen; MMTV: Mouse mammary tumor virus.

## Competing interests

The authors declare that they have no competing interests.

## Authors’ contributions

SR and LKA conceived and designed the project. MJN participated in the analysis of mammary gland development and differentiation. J.W. participated in the tumor and metastasis data analysis. WJM provided the MMTV/PyMT mouse model and participated in the study design. BTC performed some of the immunohistochemical data analysis. SR and RGK carried out the monitoring of the mice and the tissue collections. SR analysed the data and drafted the manuscript. All authors read and approved the final manuscript.

## Pre-publication history

The pre-publication history for this paper can be accessed here:

http://www.biomedcentral.com/1471-2407/14/509/prepub

## Supplementary Material

Additional file 1: Figure S1 Diagram representing the breeding protocol used to generate the F1 (FVBxB6) PyMT *Cd151*^+/+^ and *Cd151*^−/−^ experimental animals. Click here for file

Additional file 2: Figure S2 Effect of the genetic background on MMTV/PyMT tumor onset. The appearance of mammary tumors in wild-type pure FVB or F1 FVB:B6 mice was monitored by bi-weekly palpation and is represented on a Kaplan-Meier survival curve. T_50_ indicates the median time to development of a palpable mammary tumor for each group of mice. The difference in T_50_ between FVB (42 d) and F1 FVB:B6 (62.5 d) was statistically significant (*P=*0.0001). Click here for file
