# Supplementary material for: Deletion of Cd151 reduces mammary tumorigenesis in the MMTV/PyMT mouse model
Source: BMC Cancer. 2014 Jul 11;14:509. doi: 10.1186/1471-2407-14-509 (PMC4226978; doi:10.1186/1471-2407-14-509)
Supplement: Additional file 1: Figure S1 — Diagram representing the breeding protocol used to generate the F1 (FVBxB6) PyMT Cd151+/+ and Cd151−/− experimental animals. [file 1471-2407-14-509-S1.pdf]

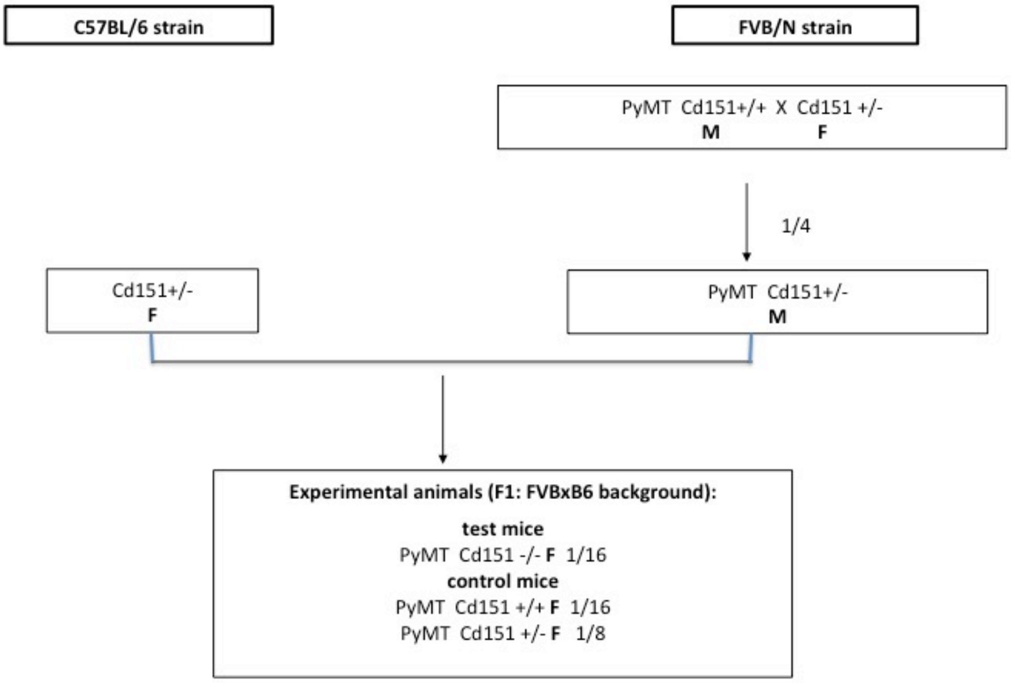

Additional file 1: Supplementary Figure 1. Diagram representing the breeding protocol used to generate the F1 (FVBxB6) PyMT *Cd151*$^{+/+}$ and *Cd151*$^{-/-}$ experimental animals.
