# Supplementary material for: Deletion of Cd151 reduces mammary tumorigenesis in the MMTV/PyMT mouse model
Source: BMC Cancer. 2014 Jul 11;14:509. doi: 10.1186/1471-2407-14-509 (PMC4226978; doi:10.1186/1471-2407-14-509)
Supplement: Additional file 2: Figure S2 — Effect of the genetic background on MMTV/PyMT tumor onset. The appearance of mammary tumors in wild-type pure FVB or F1 FVB:B6 mice was monitored by bi-weekly palpation and is represented on a Kaplan-Meier survival curve. T50 indicates the median time to development of a palpable mammary tumor for each group of mice. The difference in T50 between FVB (42 d) and F1 FVB:B6 (62.5 d) was statistically significant (P=0.0001). [file 1471-2407-14-509-S2.pdf]

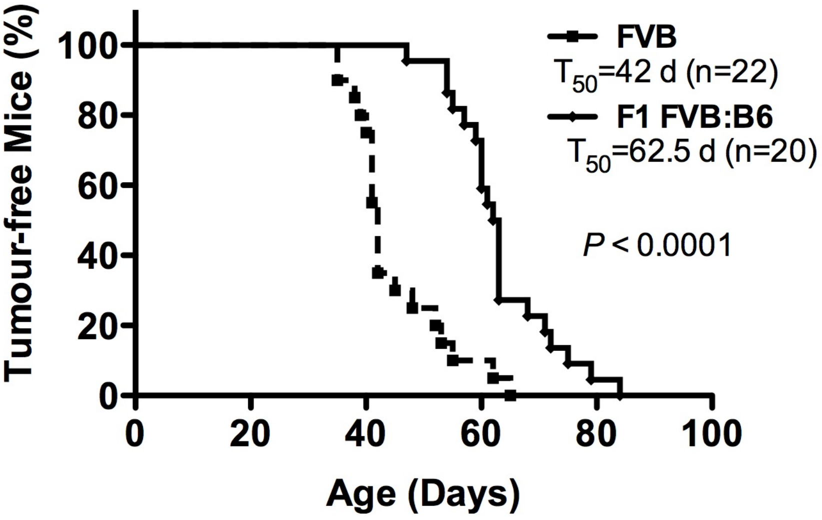

Additional file 2: Supplementary Figure 2. Effect of the genetic background on MMTV/PyMT tumor onset. The appearance of mammary tumors in wild-type pure FVB or F1 FVB:B6 mice was monitored by bi-weekly palpation and is represented on a Kaplan-Meier survival curve. T_{50} indicates the median time to development of a palpable mammary tumor for each group of mice. The difference in T_{50} between FVB (42 d) and F1 FVB:B6 (62.5 d) was statistically significant (*P*=0.0001).
